# Physiological concentrations of soluble uric acid are chondroprotective and anti-inflammatory

**DOI:** 10.1038/s41598-017-02640-0

**Published:** 2017-05-24

**Authors:** Jenn-Haung Lai, Shue-Fen Luo, Li-Feng Hung, Chuan-Yueh Huang, Shiu-Bii Lien, Leou-Chyr Lin, Feng-Cheng Liu, B. Linju Yen, Ling-Jun Ho

**Affiliations:** 1 Department of Rheumatology, Allergy and Immunology, Chang Gung Memorial Hospital, Lin-Kou, Tao-Yuan Taiwan, ROC; 2 Graduate Institute of Clinical Research, National Defense Medical Center, Taipei, Taiwan, ROC; 3 Institute of Cellular and System Medicine, National Health Research Institute, Zhunan, Taiwan, ROC; 4 Department of Orthopaedics, Tri-Service General Hospital, National Defense Medical Center, Taipei, Taiwan, ROC; 5 Rheumatology/Immunology and Allergy, Department of Medicine, Tri-Service General Hospital, National Defense Medical Center, Taipei, Taiwan, ROC

## Abstract

High uric acid levels are a risk factor for cardiovascular disorders and gout; however, the role of physiological concentrations of soluble uric acid (sUA) is poorly understood. This study aimed to clarify the effects of sUA in joint inflammation. Both cell cultures of primary porcine chondrocytes and mice with collagen-induced arthritis (CIA) were examined. We showed that sUA inhibited TNF-α- and interleukin (IL)-1β–induced inducible nitric oxide synthase, cyclooxygenase-2 and matrix metalloproteinase (MMP)-13 expression. Examination of the mRNA expression of several MMPs and aggrecanases confirmed that sUA exerts chondroprotective effects by inhibiting the activity of many chondro-destructive enzymes. These effects attenuated collagen II loss in chondrocytes and reduced proteoglycan degradation in cartilage explants. These results were reproduced in chondrocytes cultured in three-dimensional (3-D) alginate beads. Molecular studies revealed that sUA inhibited the ERK/AP-1 signalling pathway, but not the IκBα-NF-κB signalling pathway. Increases in plasma uric acid levels facilitated by the provision of oxonic acid, a uricase inhibitor, to CIA mice exerted both anti-inflammatory and arthroprotective effects in these animals, as demonstrated by their arthritis severity scores and immunohistochemical analysis results. Our study demonstrated that physiological concentrations of sUA displayed anti-inflammatory and chondroprotective effects both *in vitro* and *in vivo*.

## Introduction

Cartilage is one of the major components of synovial joints and facilitates load transfer and joint movement. Chondrocytes within the articular cartilage are responsible for synthesizing extracellular matrix (ECM)-like type II collagen and proteoglycan to maintain cartilage homeostasis^1^. Cartilage damage is the major feature of osteoarthritis (OA) and chronic inflammatory joint diseases, such as rheumatoid arthritis^2^. Many factors are responsible for and contribute to OA development; however, among these factors, inflammation is the key factor underlying OA development. The proinflammatory cytokines interleukin (IL)-1 and tumour necrosis factor alpha (TNF-α) play key roles in different types of autoimmune arthritis. In OA, IL-1 has been shown to be responsible for damaging cartilage by inducing matrix metalloproteinase (MMP) and protease activity^3^. Furthermore, the chondrocytes of OA patients express more IL-1 receptors and are more susceptible to IL-1 stimulation than other cell types^4^. Similarly, the pathogenic role of TNF-α has been well documented in several types of arthritis, and therapeutic agents targeting TNF-α have been used successfully to treat patients with different types of arthritis, such as psoriatic arthritis^5^ and rheumatoid arthritis^6^.

Uric acid is a nucleic acid degradation product whose recognized normal concentration ranges in healthy individuals are 3.4–7.2 mg/dl (200–430 mmol/l) in men and 2.4–6.1 mg/dl in women (140–360 mmol/l)^7^. Uric acid has long been considered an inert waste product, but whether it exerts any physiological effects remains poorly understood. Early studies proposed that uric acid is a signal from damaged tissues that alerts the immune system^8, 9^. For example, uric acid was shown to regulate the inflammatory response in damaged tissues in a mouse model of liver injury^10^. In addition, experimental evidence has suggested that uric acid may be important in vascular remodelling and is an independent risk factor for many vascular disorders^11^. Moreover, uric acid-lowering therapy was shown to exert beneficial effects in patients with cardiovascular diseases^12^. However, a recent study showed that administering allopurinol to heart failure patients treated with atorvastatin to reduce serum uric acid levels did not provide beneficial effects in these patients^13^.

In contrast to studies showing that elevated serum uric acid levels potentially increase the risk of cardiovascular disease, an early study suggested that uric acid may have antioxidant effects^14^. Uric acid scavenges singlet oxygen atoms and oxygen radicals, thereby attenuating iron-mediated ascorbic acid oxidation^14, 15^. Uric acid can also attenuate reperfusion damage induced by free radical-generating granulocytes in isolated organs from pigs and humans^16^. In addition, uric acid can prevent peroxynitrite-induced nitrosation of proteins and inactivation of tetrahydrobiopterin^17, 18^, a cofactor necessary for nitric oxide synthase. Increases in plasma uric acid levels are associated with reductions in plasma nitrite/nitrate levels, and treating endothelial cells with uric acid reduces vascular endothelial growth–stimulated nitric oxide (NO) production^19^.

In a mouse model of dsRNA-triggered arthritis, administering a uric acid suspension in saline reduced the frequency and severity of arthritis compared to saline treatment^20^. Interestingly, a recent study showed that uric acid concentrations in the synovial fluid of OA patients were positively correlated with the severity of knee OA^21^. Given that uric acid plays a complex role in the inflammatory response, in the present study, we investigated the possible pro- or anti-inflammatory effects of physiological concentrations (15–60 μg/ml) of soluble uric acid (sUA) in joint disease. The results of the study indicate that sUA exerts both anti-inflammatory and chondroprotective effects *in vitro* and *in vivo*.

## Results

### sUA inhibited IL-1β- and TNF-α-stimulated chondrocytes

**Figure 1:**
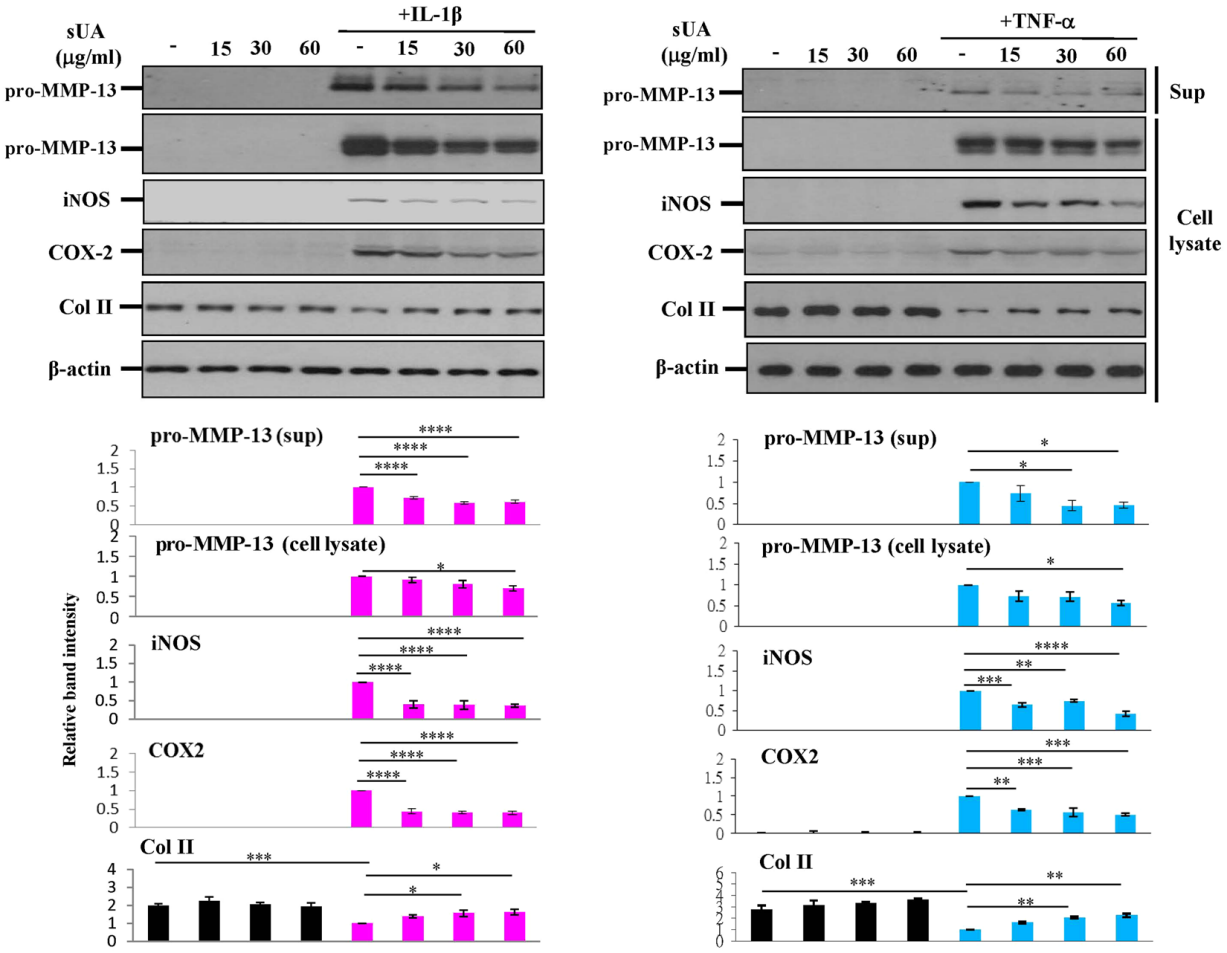
Effects of sUA on IL-1β- and TNF-α-stimulated chondrocytes. Chondrocytes (3 × 10^6^) were pretreated with different concentrations of sUA for 72 h and then stimulated with IL-1β or TNF-α for another 24 h. iNOS, COX-2, Col II, pro-MMP-13 (both in the supernatant [Sup] and in the cell lysate), and β-actin expression levels were determined by western blotting. The cropped blots are displayed and full-length blots are shown in the Supplementary Figure 7. The results are from at least 3 independent experiments. *p < 0.05; **p < 0.01; ***p < 0.001; ****p < 0.0001.

**Figure 2:**
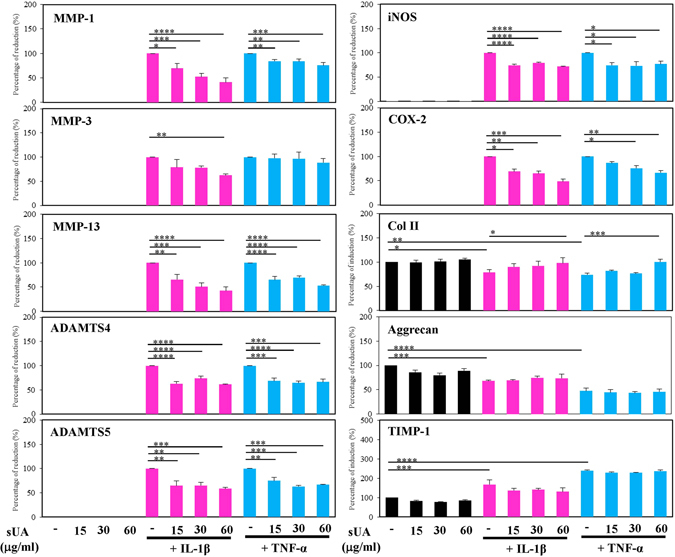
sUA suppressed TNF-α- and IL-1β-induced MMP and ADAMTS mRNA expression. Porcine chondrocytes (3 × 10^6^) in serum-free medium were treated with various concentrations of sUA for 72 h and then stimulated with 10 ng/ml IL-1β or 1 ng/ml TNF-α for an additional 24 h. MMP-1, MMP-3, MMP-13, ADAMTS4, ADAMTS5, iNOS, COX-2, Col II, aggrecan, TIMP-1, and GAPDH mRNA expression levels were determined by real-time RT-PCR. The results from at least 3 independent experiments are shown. *p < 0.05; **p < 0.01; ***p < 0.001; ****p < 0.0001.

We investigated the protective effects of several physiological concentrations of sUA on chondrocyte/cartilage degradation induced by inflammation. The results showed that IL-1β and TNF-α induced inducible nitric oxide synthase (iNOS), cyclooxygenase-2 (COX-2), and pro–MMP-13 expression in the chondrocytes, and that these effects were suppressed by sUA (Fig. 1). We consider that the incubation of cells with sUA should be as long as possible to mimic the real situation in humans. However, considering that longer incubation period may result in some unwanted conditions like contamination, we chose to pre-incubate the cells with sUA for 72 h. In very rare conditions, we also pre-incubated the cells with sUA for 24 h and the conditions also worked well. We observed that the anti-inflammatory effects of sUA could be demonstrated with as short as 6 h of pre-incubation with chondrocytes (data not shown). In addition, proinflammatory cytokine–mediated reductions in collagen II (Col II) expression were abolished by sUA treatment. We also examined the effects of monosodium urate crystals (MSU) on the above parameters. In contrast to sUA, MSU did not exert anti-inflammatory effects or reverse proinflammatory cytokine–mediated reductions in Col II expression in chondrocytes (Supplementary Figure 1). sUA also inhibited both IL-1β- and TNF-α-induced reactive oxygen species (ROS) generation (Supplementary Figure 2). To determine whether the effects of sUA are mediated by regulation of the mRNA expression levels of inflammation-related proteases and enzymes, we performed quantitative PCR assays. The primers for the genes whose expression levels were assessed by qPCR are shown in Supplementary Table 1. The results of these analyses demonstrated that sUA suppressed proinflammatory cytokine-stimulated MMP-1, MMP-13, a disintegrin and metalloproteinase with thrombospondin motifs (ADAMTS)4, ADAMTS5, iNOS and COX-2 mRNA expression in chondrocytes (Fig. 2). sUA also tended to reduce TNF-α-induced MMP-3 mRNA expression in chondrocytes, although these reductions were not statistically significant. Surprisingly, both IL-1β and TNF-α inhibited Col II mRNA expression, effects that were counteracted by sUA treatment, which also facilitated significant recovery of Col II mRNA expression levels. sUA did not affect aggrecan mRNA expression.

### Signalling pathway targeted by sUA

**Figure 3:**
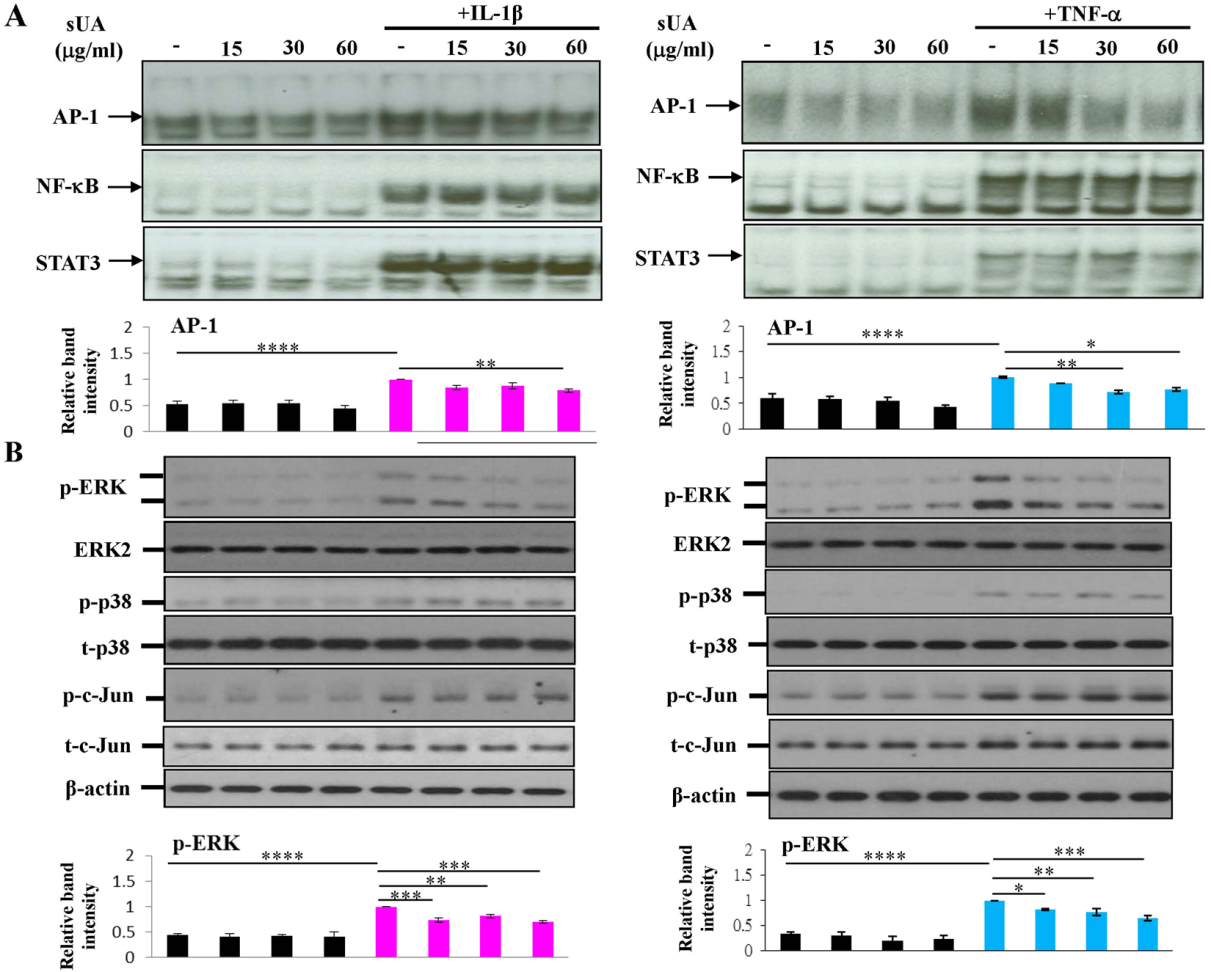
Suppression of TNF-α- and IL-1β-induced ERK-AP-1 signalling by sUA. Chondrocytes (3 × 10^6^ for each condition) were pretreated with or without various concentrations of sUA for 72 h and then stimulated with IL-1β or TNF-α for another 8 h. The cells were collected, and the nuclear extracts were prepared for the determination of AP-1, NF-κB, and STAT3 DNA-binding activity by EMSA analysis (**A**). In (**B**), the chondrocytes were pretreated with various concentrations of sUA for 72 h and then stimulated with IL-1β or TNF-α for 2 h. Phosphorylated ERK, phosphorylated p38, and phosphorylated c-Jun expression levels were determined by western blotting. The cropped blots are displayed and full-length blots are shown in the Supplementary Figure 8. The results from at least 3 independent experiments are shown. *p < 0.05; **p < 0.01; ***p < 0.001; ****p < 0.0001.

The molecular mechanisms underlying the anti-inflammatory effects of sUA were examined. As shown in Fig. 3A and Supplementary Figure 3, sUA inhibited proinflammatory cytokine-induced activator protein-1 (AP-1) but not nuclear factor kappaB (NF-κB) or signal transducer and activator of transcription (STAT)3 DNA-binding activity. These results were confirmed by reporter assays (Supplementary Figure 4A and B). In addition, neither IL-1β- nor TNF-α-mediated NF-κB inhibitor-α (IκBα) degradation was affected by sUA treatment (Supplementary Figure 4C and D). Analysis of the activity of the mitogen-activated protein kinases (MAPKs) upstream of AP-1 that are activated by IL-1β or TNF-α, i.e., the phosphorylated forms of extracellular signal–regulated kinase (ERK), p38, and c-Jun, showed that ERK, but not p38 or c-Jun N-terminal kinase (JNK), was susceptible to inhibition by sUA (Fig. 3B and Supplementary Figure 3).

### Effects of sUA in chondrocytes cultured in 3-D alginate beads

**Figure 4:**
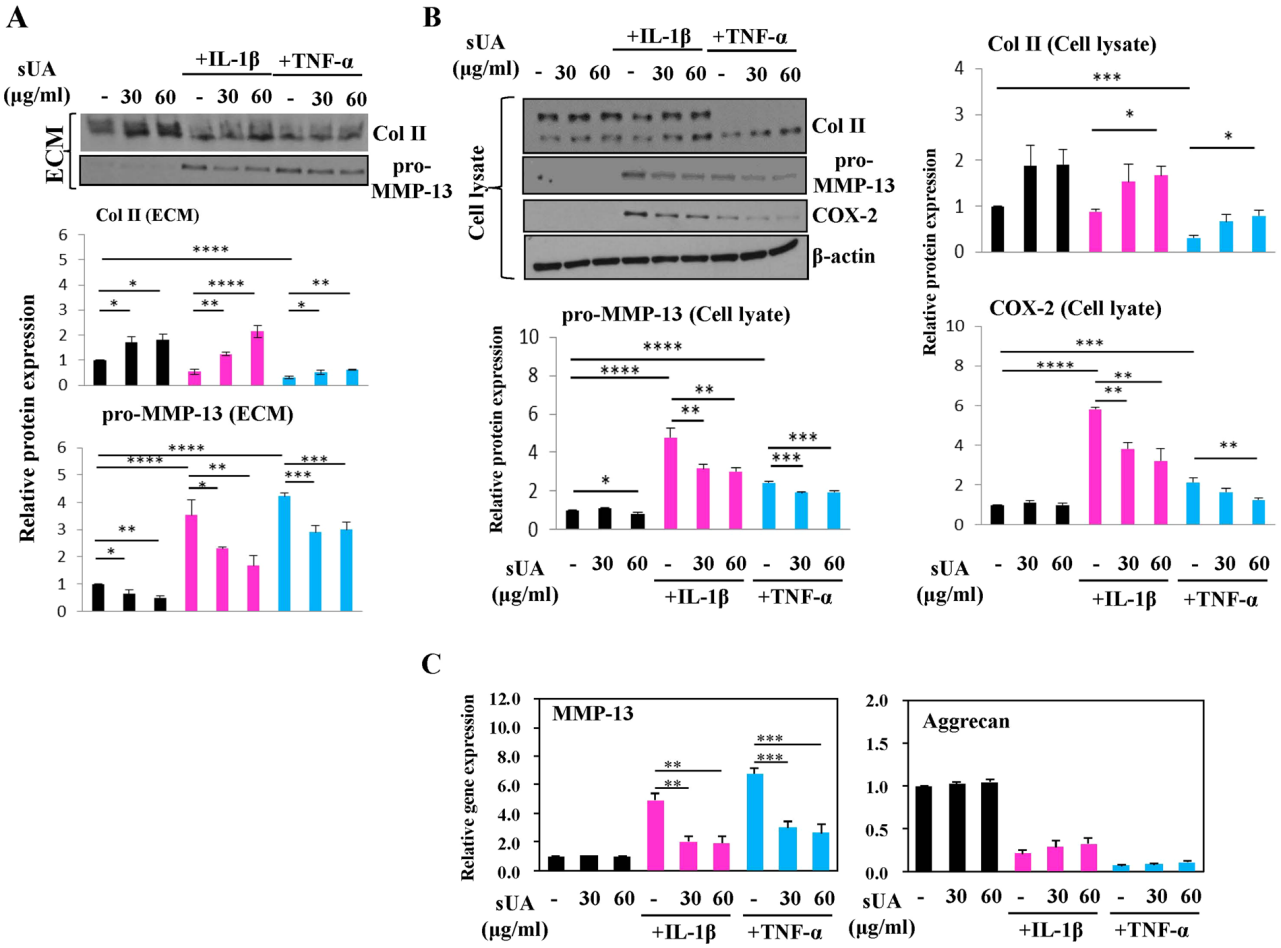
Effects of sUA on TNF-α- and IL-1β-stimulated chondrocytes cultured in alginate beads. Similar to the experiment described in Fig. 1, this experiment was performed to examine the effects of sUA on IL-1β– and TNF-α–stimulated chondrocytes in alginate beads. The results of the examination of the ECM and cellular lysates are shown in (**A**) and (**B**), respectively. MMP-13 and aggrecan mRNA expression levels were determined by real-time RT-PCR (**C**). The cropped blots are displayed and full-length blots are shown in the Supplementary Figure 9. The results from at least 3 independent experiments are shown. *p < 0.05; **p < 0.01; ***p < 0.001; ****p < 0.0001.

To avoid the confounding effects of the occurrence of chondrocyte de-differentiation in a monolayer culture, we encapsulated chondrocytes in 3-D alginate beads to ensure that the chondrocytic phenotype was retained^22^. As shown in Fig. 4A and B, sUA treatment counteracted IL-1β- and TNF-α-mediated reductions in Col II production and inhibited MMP-13 expression in samples collected from ECM and cell lysates. sUA also effectively inhibited IL-1β- and TNF-α-induced MMP-13 mRNA expression. However, sUA did not affect aggrecan mRNA expression, which served as a control in this experiment (Fig. 4C).

### sUA protected against TNF-α- and IL-1β-induced proteoglycan degradation in cartilage explants

**Figure 5:**
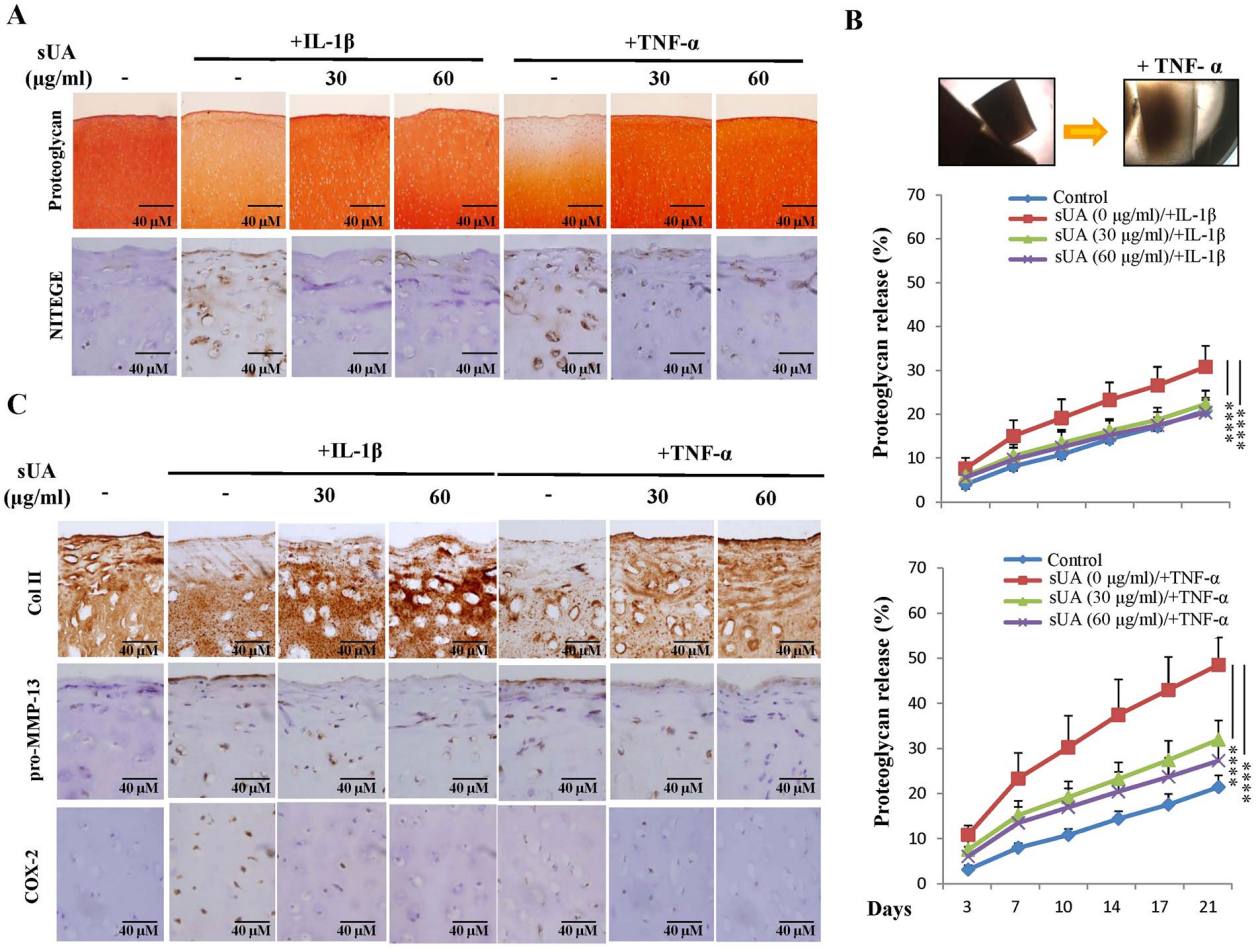
Effects of sUA on TNF-α- and IL-1β-mediated damage in cartilage explants. Each piece (3 mm in diameter) of porcine cartilage fragment was cultured in a 24-well plate with DMEM containing antibiotics and 10% FCS. The cartilage explants were pretreated with various concentrations of sUA for another 72 h and then stimulated with IL-1β or TNF-α. The retained proteoglycan, which displayed positive safranin O staining, and the neoepitope generated by ADAMTS cleavage in the cartilage explants was monitored by immunohistochemical staining (**A**). Untreated and TNF-α-treated cartilage explants were observed by microscope (**B**, upper panel). Proteoglycan release into the culture medium or retained in the cartilage explant was determined by DMB assays (**B**, lower panel). (**C**) Shows the results of staining for Col II, pro-MMP-13, and COX-2 expression. Six blocks were included for each condition, and the results from 5 independent experiments are shown. *p < 0.05; **p < 0.01; ***p < 0.001; ****p < 0.0001.

To further investigate the chondroprotective effects of uric acid and elucidate the events associated with proteoglycan degradation in cartilage, we prepared and examined porcine cartilage explants of equal sizes. As shown in Fig. 5A, sUA prevented both IL-1β- and TNF-α-induced proteoglycan loss and inhibited both IL-1β- and TNF-α-enhanced NITEGE, the carboxyl-terminal aggrecan cleavage product, staining in cartilage explants. Furthermore, sUA suppressed IL-1β- and TNF-α-mediated release of proteoglycan into the culture supernatants of cartilage explants (Fig. 5B). Immunohistochemical staining for COX-2, MMP-13, and Col II confirmed that sUA exerts chondroprotective effects in proinflammatory cytokine-induced inflammation and cartilage damage (Fig. 5C).

### Effect of hyperuricaemia in a murine CIA model

**Figure 6:**
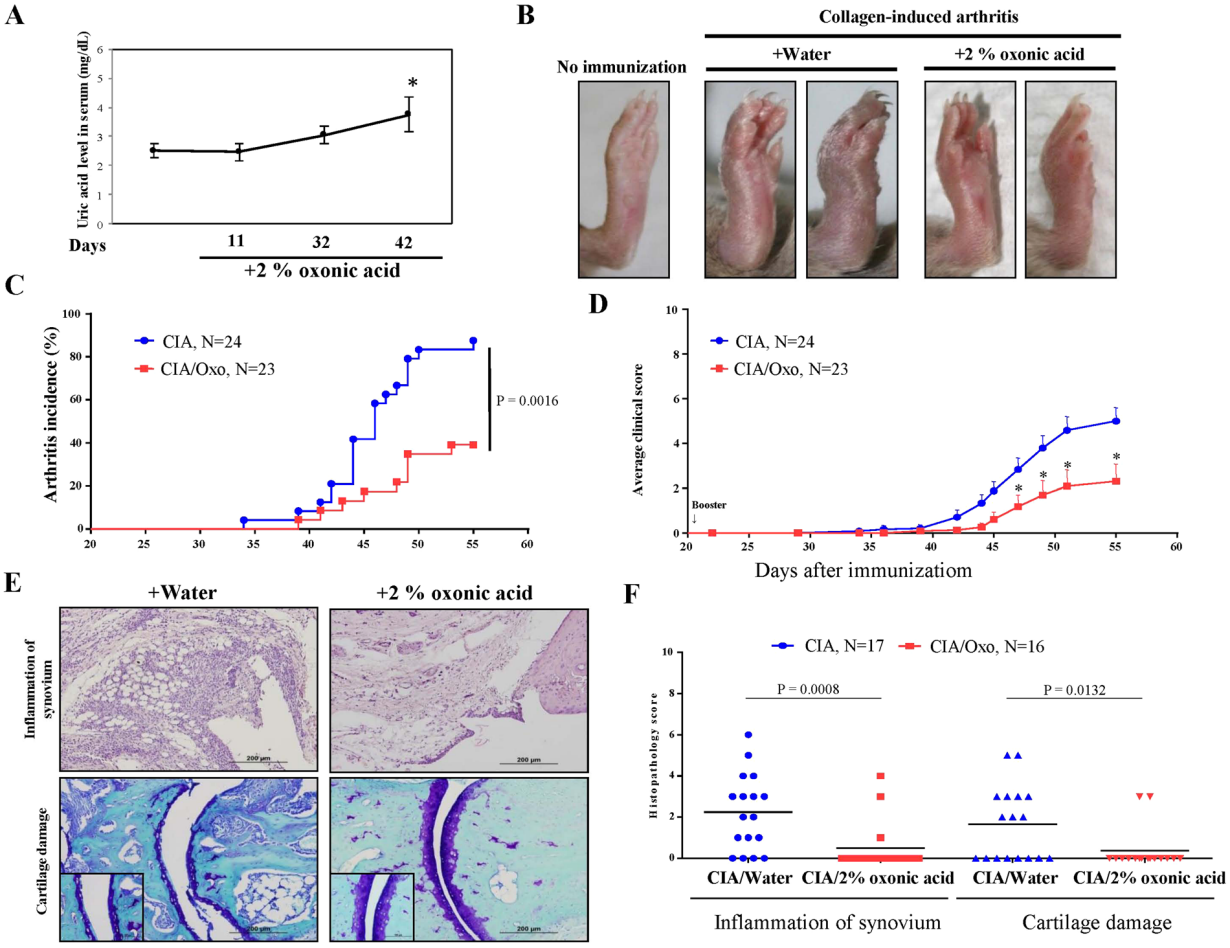
Effects of sUA on mice with collagen-induced arthritis. Three individual independent experiments were conducted sequentially, and the results of the experiments were pooled for analysis. Each independent experiment comprised solvent (water)-treated and oxonic acid-treated mice (average, 8–9 animals each). (**A**) Shows the relationship between serum uric acid concentrations and the interval after oxonic acid treatment. (**B**) Shows representative images of arthritis in water- and oxonic acid-treated mice. The incidence of arthritis (**C**) and average clinical scores (**D**) were calculated for the control and oxonic acid-treated groups. The results of the immunohistochemical analysis of joint tissue specimens were analysed. (**E**) Shows representative images of synovial inflammation and cartilage damage, and (**F**) shows the histopathology scores. The numbers of animals involved in individual analyses are shown.

**Figure 7:**
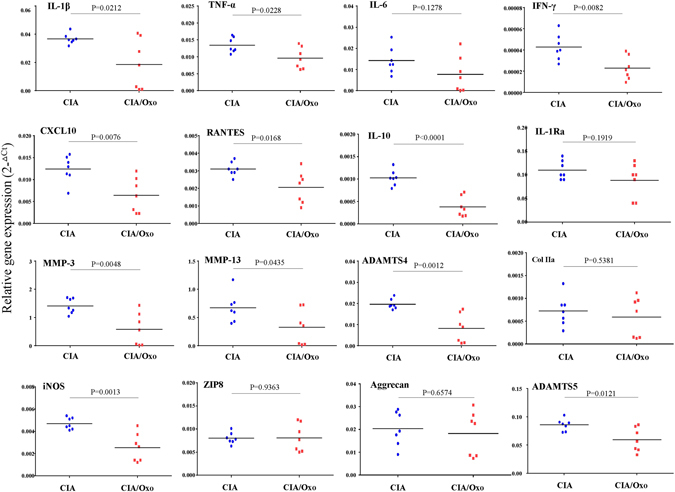
sUA regulated the mRNA expression of proinflammatory cytokines, chemokines, and several proinflammatory markers in the inflamed joints of CIA mice. The tissues taken from the entire joint/paw of water- and oxonic acid-treated CIA mice (7 for each) were collected, and the mRNA expression levels of several proinflammatory cytokines, chemokines, and inflammation-related molecules were analysed, as indicated previously. The primers for the individual genes whose expression levels were measured by qPCR are shown in Supplementary Table 1.

**Figure 8:**
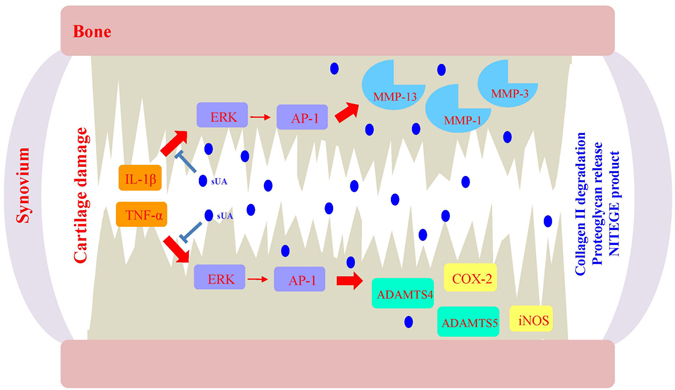
Illustration of the chondroprotective effects of sUA and the mechanisms underlying these effects. In joint inflammation involving both IL-1β and TNF-α, numerous signalling pathways and cartilage-damaging enzymes are activated. The inhibitory effects of sUA on the ERK-AP-1 signalling pathway were observed at a wide range of physiological concentrations of sUA. The ERK-AP-1 pathway targeted by sUA likely contributes to the activation of cartilage-damaging enzymes, such as MMP and ADAMTS, and inflammation-related molecules, such as iNOS and COX-2.

To investigate the possible protective effect of hyperuricaemia in the murine CIA model, the animals were fed with water or—to induce hyperuricaemia—oxonic acid, a uricase inhibitor effective in increasing serum uric acid levels^23^ (Fig. 6A). We induced polyarthritis by injecting bovine Col II into the tails of the mice. Administration of oxonic acid did not cause changes in body weight (data not shown). Based on a previously established scoring system^24^, oxonic acid-fed mice exhibited a lower incidence of arthritis and less severe arthritis than water-fed mice (Fig. 6B–D). Immunohistochemical analysis was performed to measure structural damage severity, as described by other researchers^25^ (Supplementary Figure 5). Figure 6E shows that uric acid exerted arthroprotective effects against arthritis in oxonic acid-treated mice, as these mice displayed less inflammatory cell infiltration in the synovium, less synovial hyperplasia, less cartilage damage and less bone erosion than control mice (the higher magnification images are shown in Supplementary Figure 6). The statistical data regarding the severity of both the synovial inflammation and the cartilage damage displayed by oxonic acid-treated and control animals were analysed (Fig. 6F). The results of the analysis suggest that a high correlation exists between the histological findings characterizing inflamed joint tissues and arthritis clinical data. Furthermore, increases in uric acid differentially regulated the mRNA levels of several cytokines, including IL-1β, TNF-α, IL-6, IL-10, IL-1 receptor antagonist (IL-1Ra) and IFN-γ, as well as the levels of chemokines, such as CXCL10 and regulated on activation, normal T cell expressed and secreted (RANTES), and the levels of cartilage-damaging enzymes, including MMP-3, MMP-13, ADAMTS4, ADAMTS5, and iNOS, in CIA mice (Fig. 7). Oxonic acid treatment did not affect the mRNA expression of ZIP8, a Zn2+ importer capable of inducing the expression of several MMPs and ADAMTS5 in OA^26^. The anti-inflammatory and chondroprotective effects of sUA and the mechanisms underlying these effects are summarized in Fig. 8.

## Discussion

An early study published a few decades ago reported that sUA has no effect on chondrocyte viability, proliferation or proteoglycan synthesis^27^. No other studies have examined the effects of sUA at physiological concentrations on chondrocytes. Furthermore, many researchers have long considered plasma sUA a waste product. This study therefore aimed to clarify the roles of sUA in cartilage and joint inflammation. Although above-normal uric acid levels may be risk factors for several diseases^28^, our results demonstrate that physiological concentrations of uric acid exert anti-inflammatory and chondroprotective effects. These effects were clearly demonstrated using many different cellular and molecular approaches in different systems, including a chondrocyte-based study, a 3-D alginate bead study and study using cartilage explants.

ECM components, such as collagen, proteoglycan and aggrecan, form structural bases that are essential for maintaining cartilage integrity^29^. Among the many types of collagen, Col II is particularly important and has long served as an accurate indicator of cartilage metabolism^30^. MMP-13 (collagenase-3) preferentially cleaves Col II with greater potency than collagenase-1 and is a critical proteinase in cartilage damage, as well as in progressive cartilage matrix and cellularity loss in the ageing process^31, 32^. Many other MMPs and aggrecanases, such as the ADAMTSs, also have roles in cartilage damage in inflamed joints^33^. These results suggest that many of these proinflammatory cytokine-induced chondro-destruction-inducing enzymes can be effectively suppressed under physiological sUA concentrations. Surprisingly, in addition to downregulating chondro-destruction-inducing enzymes, sUA also inhibited proinflammatory cytokine-mediated suppression of Col II mRNA expression. It is therefore possible that sUA targets more upstream molecules in the signalling pathways involved in proinflammatory cytokine stimulation.

Molecular analysis revealed the specificity of the chondroprotective effects of sUA. Among the MAPK pathways, the ERK signalling pathway, but not the p38- or JNK-mediated signalling pathway, was affected by sUA. Similarly, the AP-1-mediated signalling pathway but not the NF-κB signalling pathway was targeted by sUA. These results are consistent with those of a previous study that showed that AP-1 activation plays an important role in MMP-13 expression^34^. Furthermore, given the critical role played by AP-1 family proteins in the mediation of OA cartilage destruction, it is possible that sUA-mediated AP-1 signalling pathway inhibition can protect against OA pathogenesis^35^. The AP-1 signalling pathway-selective character of sUA shares certain similarities with the anti-inflammatory effects of retinoic acid observed in previous studies^36^. Although our studies demonstrated that sUA could inhibit proinflammatory cytokine-induced ERK activation, these experiments did not identify the exact target that is inhibited by sUA. That is, the current study did not show whether sUA directly or indirectly inhibited the ERK/AP-1 signalling pathway. Further studies are needed to address this issue.

For decades, there have been no satisfactory animal models of OA. The commonly used traumatic OA model, which is induced by anterior cruciate ligament transection, is representative of only a very limited number of OA populations. Because the cartilage damage in OA and rheumatoid arthritis is similar^2^, we investigated the anti-inflammatory effects of sUA in a murine model of CIA. We noted significant changes in synovitis development, cartilage destruction and bone erosion, changes indicating that the CIA model is a very useful animal model of arthritis, especially rheumatoid arthritis^37^. In this model, many cytokines, such as IL-1β, TNF-α, IL-6 and IL-10, play important roles in the pathogenesis of the disease^37^. The uricase inhibitor oxonic acid, which increases plasma uric acid levels, reduced the incidence and severity of arthritis in CIA mice. We also noted significant reductions in the levels of proinflammatory cytokines, such as IL-1β and TNF-α, as well as suppression of chemokine and cartilage destruction-inducing enzyme production, in oxonic acid-treated mice compared to control mice. The cumulative effects of these changes also led to reduced inflammatory cell infiltration into the synovial tissues, as well as protection against cartilage damage and bone erosion. The anti-inflammatory roles of IL-10^38, 39^ and IFN-γ^40, 41^ have been established; our results showed that uric acid also reduced both IL-10 and IFN-γ expression in inflamed joints. However, these reductions did not have significant impact on the chondroprotective and anti-inflammatory effects of sUA. Meanwhile, the mRNA levels of the proinflammatory cytokine IL-6 and the anti-inflammatory cytokine IL-1Ra also decreased after oxonic acid treatment, although the results of our statistical analysis indicated that the changes were not significant due to the limited number of samples analysed. Overall, the results of our *in vivo* studies using CIA mice fully support the results of our *in vitro* studies using primary chondrocytes.

sUA has been shown to exert anti-inflammatory effects in patients with acute knee injuries^42^. Furthermore, an extensive meta-analysis that compared the incidence of bone mineral density, osteoporosis and fractures in people with higher serum uric acid concentrations to those in people with lower serum uric acid concentrations revealed that uric acid plays a protective role. Moreover, subjects with higher serum uric acid levels have a significantly higher bone mineral density than subjects with lower serum uric acid levels^43^. Consistent with the results of these human studies, our results showed that physiological serum sUA levels exerted anti-inflammatory effects that protected against inflammation-induced cartilage and joint damage.

## Methods

### Reagents and antibodies

sUA solution was freshly prepared and filtered through a 0.22-μm syringe filter, as described previously^44^. MSU was prepared using the method reported by Lee *et al*.^45^. Recombinant TNF-α and IL-1β were purchased from R&D Systems, Inc. (St. Paul, MN, USA). Polyclonal antisera against total ERK-1, ERK-2, p38, and c-JNK were obtained from Santa Cruz Biotechnology (Santa Cruz, CA, USA), and antibodies recognizing phosphorylated ERK, phosphorylated p38, and phosphorylated JNK were purchased from Cell Signaling Technology, Inc. (Beverly, MA, USA). Polyclonal antisera against MMP-13, iNOS and COX-2 were purchased from Santa Cruz Biotechnology (Santa Cruz, CA). Polyclonal anti–Col II antibodies were purchased from Chemicon International (Temecula, CA), and antibodies recognizing NITEGE were purchased from Novus Biologicals. Unless otherwise specified, all other reagents were purchased from Sigma-Aldrich Chemical Company (St. Louis, MO, USA).

### Isolation and culture of porcine chondrocytes

Porcine cartilage specimens were obtained from the hind leg joints of pigs. Chondrocytes were prepared from these specimens as described in our previous report^46^. After the articular cartilage was enzymatically digested with 2 mg/ml protease in serum-free Dulbecco’s modified Eagle’s medium (DMEM) containing antibiotics and 10% foetal bovine serum (FBS), the specimens were digested overnight with 2 mg/ml collagenase I and 0.9 mg/ml hyaluronidase in DMEM/antibiotics. The cells were subsequently collected, passed through a cell strainer (Beckton Dickinson, Mountain View, CA, USA) and cultured in DMEM containing 10% FBS and antibiotics for 3–4 days before being used. When cultured in a monolayer, chondrocytes de-differentiate into fibroblast-like cells after a few passages^47–49^. To prevent this change, we maintained the chondrocytes used throughout this study at one passage so that the cells retained their shapes and characteristics^50–52^. During the period of cell culture and treatment, sUA was not removed.

### Western blotting

Enhanced chemiluminescence Western blotting (Amersham-Pharmacia, Arlington Heights, IL, USA) was performed as described previously^52^. Briefly, equal amounts of protein were analysed using sodium dodecyl sulfate–polyacrylamide gel electrophoresis (SDS-PAGE) and transferred to a nitrocellulose filter. For immunoblotting, the nitrocellulose filter was incubated with Tris-buffered saline with 1% Triton X-100 containing 5% non-fat milk for 1 h and then blotted with antibodies against specific proteins for another 2 h at room temperature.

### Nuclear extract preparation and electrophoretic mobility shift assay

Nuclear extract preparation and electrophoretic mobility shift assay (EMSA) were performed as described in our previous report^52^. Oligonucleotides containing an NF-κB-, STAT3-, or AP-1-binding site were purchased and used as DNA probes. The DNA probes were radiolabelled with [γ-^32^P]ATP using T4 kinase (Promega). For the binding reaction, the radiolabelled probe was incubated with 4 μg of nuclear extracts. The binding buffer contained 10 mM Tris-HCl (pH 7.5), 50 mM NaCl, 0.5 mM ethylenediaminetetraacetic acid (EDTA), 1 mM dithiothreitol, 1 mM MgCl_2_, 4% glycerol, and 2 μg of poly(dI-dC). The final reaction mixture was analysed in a 6% non-denaturing polyacrylamide gel, and 0.5× Tris/Borate/EDTA was used as an electrophoresis buffer.

### Analysis by real-time polymerase chain reaction with reverse transcription

Total RNA was isolated using Trizol reagent (Invitrogen; Carlsbad, CA, USA) after the cells were lysed, according to the manufacturer’s protocol and as described in our previous report^52^. Reverse transcription was performed in a 20-μl mixture containing 2 μg of total RNA, 10× RT buffer (Invitrogen), random hexamers (Invitrogen), a mixture of dNTP (Promega; Madison, WI, USA), and Moloney Murine Leukemia Virus Reverse Transcriptase (MMLV RTase, Invitrogen), in accordance with the protocol governing the use of the Superscript First-Strand Synthesis System (Invitrogen). After the RNA was reverse-transcribed to cDNA, the obtained template cDNA samples were subjected to PCR reactions. Real-time measurements of the expression levels of the designated genes were performed according to the manufacturer’s instructions (power SYBR Green PCR Master Mix, Applied BioSystems, Foster City, CA, USA). Briefly, 10 ng of cDNA was amplified in a total mixture volume of 20 μl consisting of 1× Master Mix and the appropriate gene-specific primers, which were added at a final concentration of 100 nM. The primer sequences, which are shown in Supplementary Table 1, were designed by us or described by other researchers^53, 54^. The reactions were performed over 40 cycles comprising steps at 95 °C for denaturation and 60 °C for annealing and extension on a Roche LightCycler 480 (Roche). The changes in gene expression caused by stimulation with TNF-α or IL-1β in the presence or absence of sUA were calculated with the following formula: fold change = 2^−Δ(ΔC^_t_^)^, where ΔC_t_ = C_t stimulated_ − C_t GAPDH_, and Δ(ΔC_t_) = ΔC_t stimulated_ −ΔC_t control_.

### Transfection assays

Transient transfection was performed using the transfection reagent TransIT-LT1 (Mirus Bio LLC, Madison, WI, USA). Briefly, chondrocytes at P0 were transfected with a DNA/TransIT-LT1 preparation in 10% FBS culture medium. The transfection mixture consisted of 15 μg of AP-1 or NF-κB firefly luciferase reporter plasmid (Stratagene, La Jolla, CA, USA), 1 μg of the internal control plasmid TK-Renilla luciferase (Promega, Madison, WI, USA) and 45 μl of TransIT-LT1 in Opti-MEM. The chondrocytes were passaged in a 24-well plate at a density of 4 × 10^5^/well overnight, after which the medium was replaced with serum-free DMEM containing various concentrations of sUA. After 72 h, the cells were treated with IL-1β or TNF-α for another 24 h before the total cell lysate was collected, and luciferase activity was measured using a luminometer, according to the manufacturer’s instructions (Promega). Renilla luciferase values were used to normalize each sample for transfection efficiency measurements. The results are expressed as fold inductions of luciferase activity.

### 3-D alginate bead experiments

The 3-D alginate bead experiments were performed by slightly modifying a previously described method^55^. Briefly, freshly isolated chondrocytes were gently resuspended in alginate solution (1.2% low-viscosity alginate in 0.15 M NaCl) at a density of 3.75 × 10^6^ cells/ml. The chondrocyte suspension was slowly dripped (drop volume, 10 μl) into a CaCl_2_ solution (102 mM) using an automatic Pipetman. After the solution had slowly mixed, and the beads had been allowed to completely polymerize for 10 min at room temperature, the CaCl_2_ solution was aspirated, and the beads were washed with normal saline before being cultured in DMEM containing 10% FCS at 37 °C with 5% CO_2_. After the beads were treated for the indicated time period, the culture medium was replaced with iced normal saline. The alginate beads were then transferred into Eppendorf tubes containing a cold 55 mM sodium citrate solution and rotated for 30 min at 4 °C to dissolve the alginate gel and release the cells from the beads. After the cells were centrifuged at 12,000 g for 10 min at 4 °C, the supernatants (ECM fraction) were collected. The cell pellet was washed with cold normal saline and lysed in RIPA buffer. The ECM fraction and cell lysate were then analysed with the relevant assays.

### Preparation of cartilage explants

The cartilage explants were performed as described in our previous report^52^. Briefly, articular cartilage specimens of uniform size from the joint located near the femoral head of the pig hind-limb were excavated by a stainless-steel dermal-punch (diameter, 3 mm; Aesculap, Tuttlingen, Germany) and weighed. Each cartilage explant was subsequently placed in a 96-well plate and cultured in DMEM containing antibiotics and 10% FBS for 24 h. After incubating in serum-free DMEM for 72 h, the cartilage explants were used for additional experiments.

### Analysis of cartilage degradation

We assessed cartilage degradation by measuring the amount of proteoglycan released into the culture medium, as previously described^52^. Briefly, culture medium was added to 1,9-dimethylmethylene blue (DMB) solution (Sigma), which comprises a metachromatic dye that can bind sulfated glycosaminoglycan (GAG), a major component of proteoglycan. GAG-DMB complex formation was quantified in a 96-well plate using a plate reader (TECAN) at a wavelength 595 nm (released GAG). The cartilage explants were then collected into Eppendorf tubes and digested in papain (1 mg/ml containing 5 mM cysteine HCL, 5 mM EDTA, and 0.1 M phosphate buffer, pH 6.0) at 60 °C overnight. The dissolved matrix was subsequently analysed to determine the proteoglycan content of each sample (retained GAG). GAG loss was calculated and was expressed as [the released GAG (μg) in culture medium/the released GAG + the retained GAG in cartilage explant] × 100.

### Safranin O staining and immunohistochemical study

Cartilage explants were mounted in embedding medium (Miles Laboratories, Naperville, IL, USA) and rapidly frozen at −80 °C, and then serial, noncontiguous microscopic sections (7 μm) of cartilage explants were cut on a Microm cryostat at −20 °C and mounted on Superfrost Plus glass slides (Menzel-Gläser, Braunschweig, Germany). To assess changes in proteoglycan content, we stained the tissue sections with safranin O/fast green before counterstaining the tissues with Weigert’s iron haematoxylin^52^. We then performed immunohistochemical staining to assess pro-MMP-13, Col II and COX-2 expression and performed NITEGE as described in our previous report, with some modifications^50^.

### Animal experiments

All animal experiments performed in this study were approved by the National Health Research Institute, Taiwan, and all mice were housed and maintained under specific pathogen-free conditions, according to the institute’s animal care guidelines. In addition, all methods in animal studies were performed in accordance with the relevant guidelines and regulations of the institution. The murine CIA model was produced as previously described^24^. The tails of male DBA/1 J mice (age, 9 weeks) were injected intradermally with 100 μl of bovine Col II at a concentration of 1 mg/ml and complete Freund’s adjuvant containing 0.5 mg/ml of *Mycobacterium tuberculosis*. The animals received a 50-μl booster injection 21 days after the first injection. Twenty-eight days after Col II injection, the animals were closely observed every 2–3 days to determine any inflammatory reactions had occurred in their foot paws. The following 4-point scale was used to measure clinical disease activity in each paw: 0 = No evidence of erythema and swelling; 1 = Erythema and mild swelling confined to the tarsals or ankle joint; 2 = Erythema and mild swelling extending from the ankle to the tarsals; 3 = Erythema and moderate swelling extending from the ankle to the metatarsal joints; and 4 = Erythema and severe swelling encompassing the ankle, foot and digits or limb ankylosis^24^. The mice were sacrificed via CO_2_ inhalation 56 days later, after which their blood was aspirated from their hearts, and their serum was collected (3000 × g, 15 min at 4 °C) for analysis of their uric acid concentrations. The foot paw samples were immersed in 10% formalin and fixed for pathological analysis after haematoxylin/eosin staining and toluidine blue O staining or stored in liquid nitrogen for mRNA expression analysis. Pathological inflammatory changes and cartilage destruction in joints were scored using a previously described classification system, with some modifications^25, 56^. The following 3-point scale for synovial inflammation was used in the study: 0 = Normal; 1 = Minimal inflammatory cell infiltration into the synovium; 2 = Moderate inflammatory cell infiltration into the synovium, synovial hyperplasia and oedema; and 3 = Severe diffuse infiltration, pannus formation and severe oedema. The following 3-point scale for cartilage degradation measurement was used in the study: 0 = Normal; 1 = mild loss of toluidine blue O staining in the superficial layer and slight surface fibrillation; 2 = moderate loss of toluidine blue O staining and cartilage disruption; and 3 = severe loss of toluidine blue O staining, complete loss of cartilage and bone erosion. The liquid nitrogen–preserved samples were ground with a pestle in SPEX SamplePrep 6770 Freezer/Mill and dissolved in Trizol to obtain RNA. After the RNA was reverse transcribed into cDNA, mRNA expression levels were determined. The mice in the treatment group were fed 2% oxonic acid (Sigma-Aldrich) in reverse osmosis-treated water to induce hyperuricaemia, as previously described^57^.

### Statistical analysis

One-way ANOVA with Bonferroni’s multiple comparison test was used for multiple comparisons, and Student’s t-test was used to evaluate differences between groups. To measure arthritis incidences and histopathology scores, we performed chi-square contingency analysis and nonparametric Mann-Whitney U-tests, respectively. P values less than 0.05 were considered significant (*p < 0.05; **p < 0.01; ***p < 0.001, ****p < 0.0001).

## Electronic supplementary material

Physiological concentrations of soluble uric acid are chondroprotective and anti-inflammatory
