# Supplementary material for: Physiological concentrations of soluble uric acid are chondroprotective and anti-inflammatory
Source: Sci Rep. 2017 May 24;7:2359. doi: 10.1038/s41598-017-02640-0 (PMC5443811; doi:10.1038/s41598-017-02640-0)
Supplement: Supplementary file 1 — Physiological concentrations of soluble uric acid are chondroprotective and anti-inflammatory [file 41598_2017_2640_MOESM1_ESM.doc]

**[Supplementary information](http://journals.bmj.com/site/authors/preparing-manuscript.xhtml#supplementary)**

**Title:** Physiological concentrations of soluble uric acid are chondro-protective and anti-inflammatory

**Authors:** Jenn-Haung Lai, Shue-Fen Luo, Li-Feng Hung, Chuan-Yueh Huang, Shiu-Bii Lien, Leou-Chyr Lin, Feng-Cheng Liu, B Linju Yen, and Ling-Jun Ho

Supplementary Table 1.


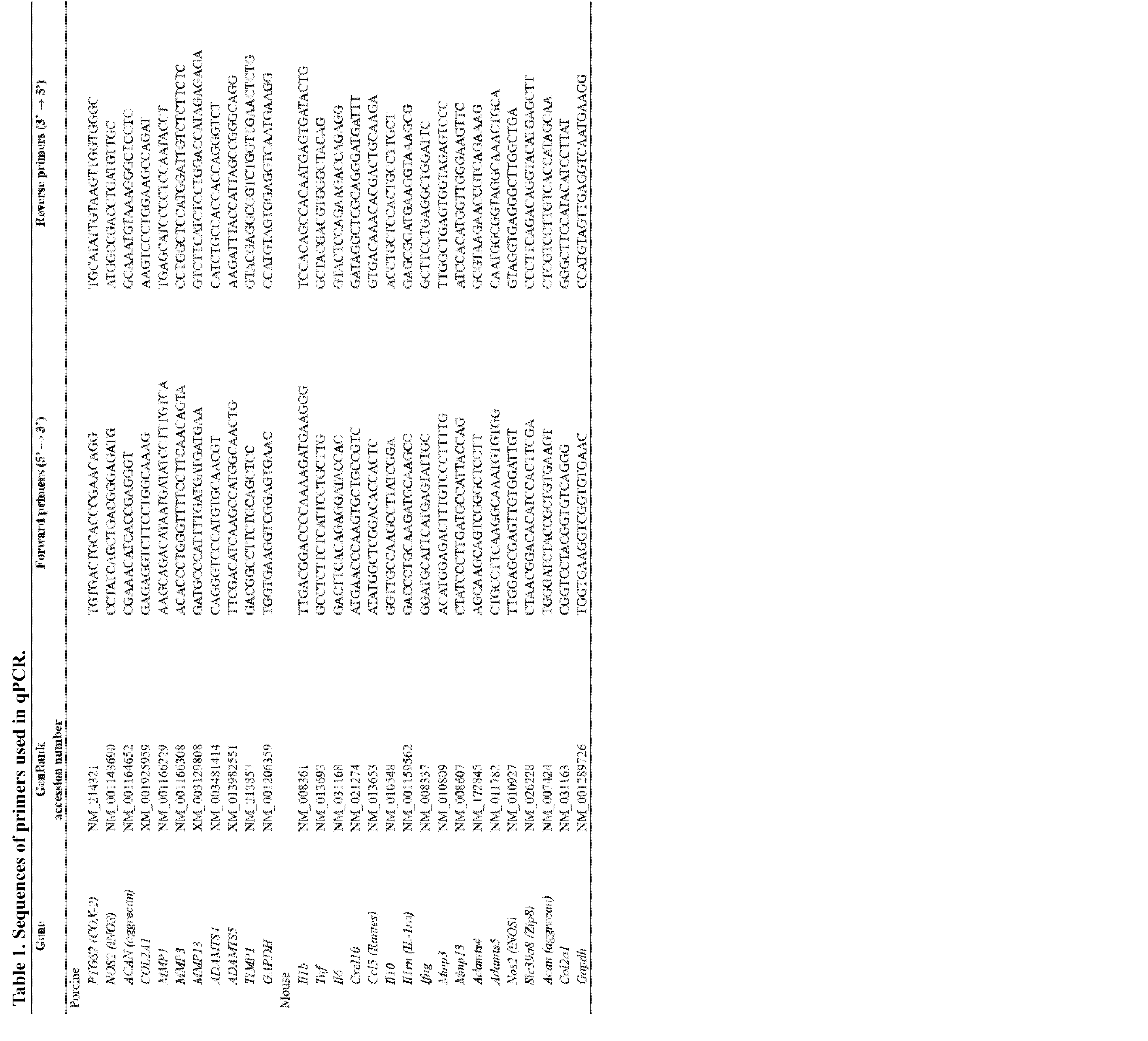


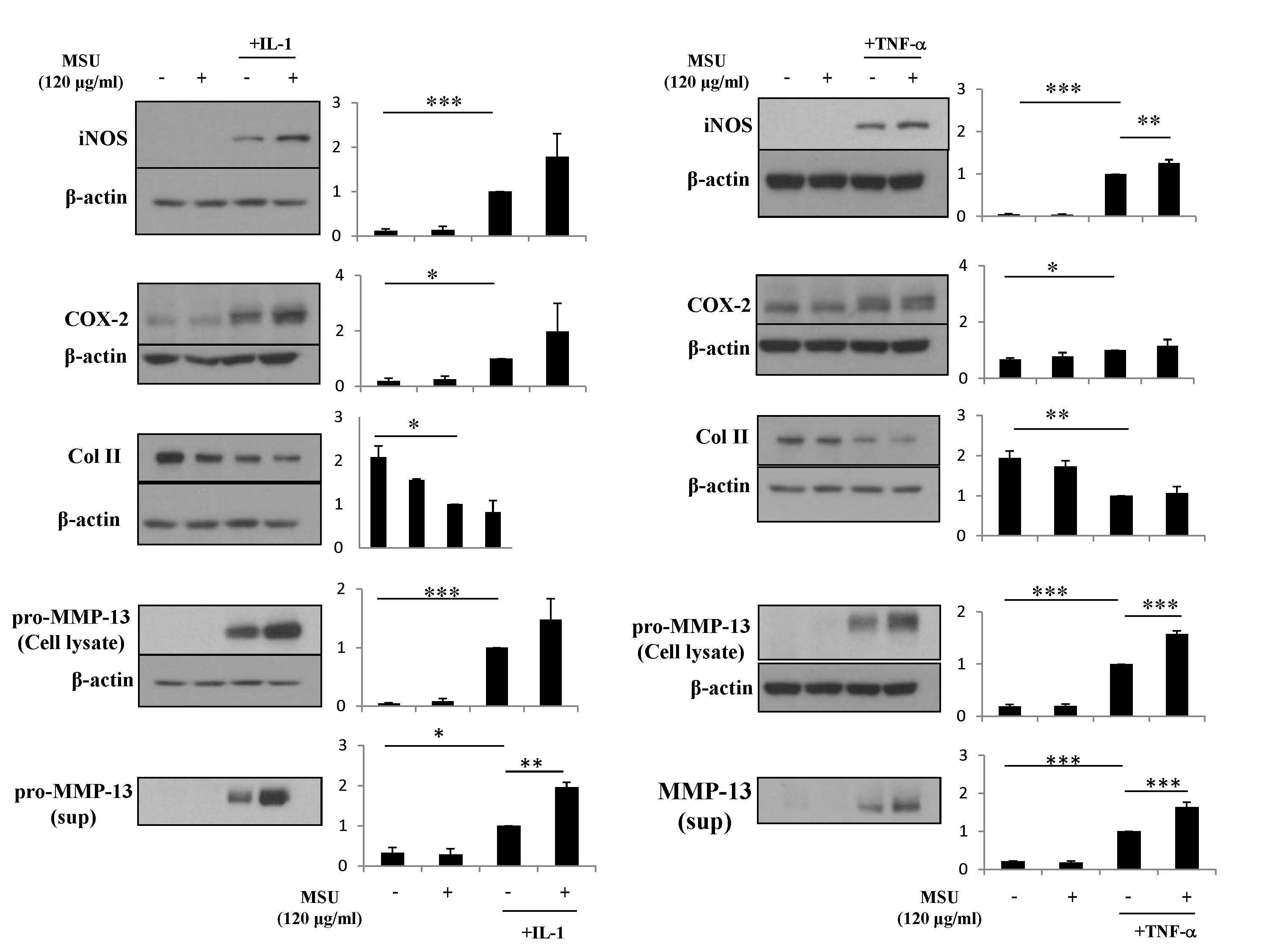


**Supplementary figure 1** Effects of MSU crystals on TNF-α– and IL-1–induced iNOS, COX-2, Col II, and pro-MMP-13 in porcine chondrocytes. Porcine chondrocytes (3 × 10^6^) were pretreated with MSU crystals at 120 μg/ml for 24 h and then stimulated with IL-1 or TNF-α for another 24 h. Expressions of iNOS, COX-2, Col II, pro-MMP-13 (in supernatant [Sup] and cell lysate), and β-actin were determined by Western blot. The densitometric intensity of individual bands was measured and compared with the stimulated samples in the absence of MSU, after normalization to internal β-actin density. The representative results of at least 3 independent experiments using different donor cells are shown. *p < 0.05; **p < 0.01; ***p < 0.001.


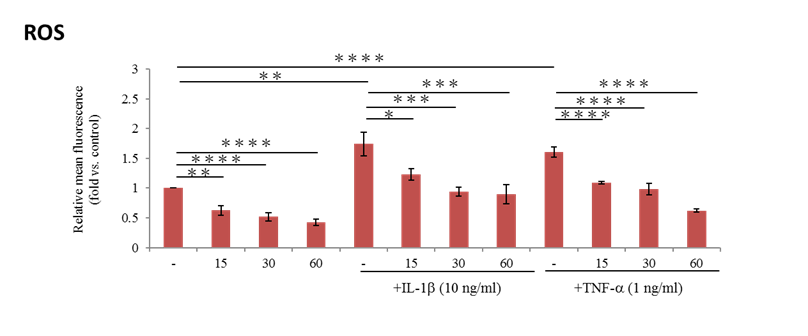


**Supplementary figure 2** Effects of sUA on ROS production induced by TNF-α and IL-1 in porcine chondrocytes. Porcine chondrocytes cultured in 24 well-plate and exposed to sUA for 72 hr were stimulated with IL-1β or TNF-α for another 24 hr. The free radical sensor CM H2DCFDA (ThermoFisher Scientific, Waltham, MA USA) at a final concentration of 2 μM was added and incubate for 4 hr before read by EnSpire® Multimode Plate Reader (PerkinElmer, Waltham, MA, USA) at Ex/Em: 485/520 nm according to the description from the manufacturers. The statistical results are from 3 independent experiments. *p < 0.05; **p < 0.01; ***p < 0.001; ****p < 0.0001.


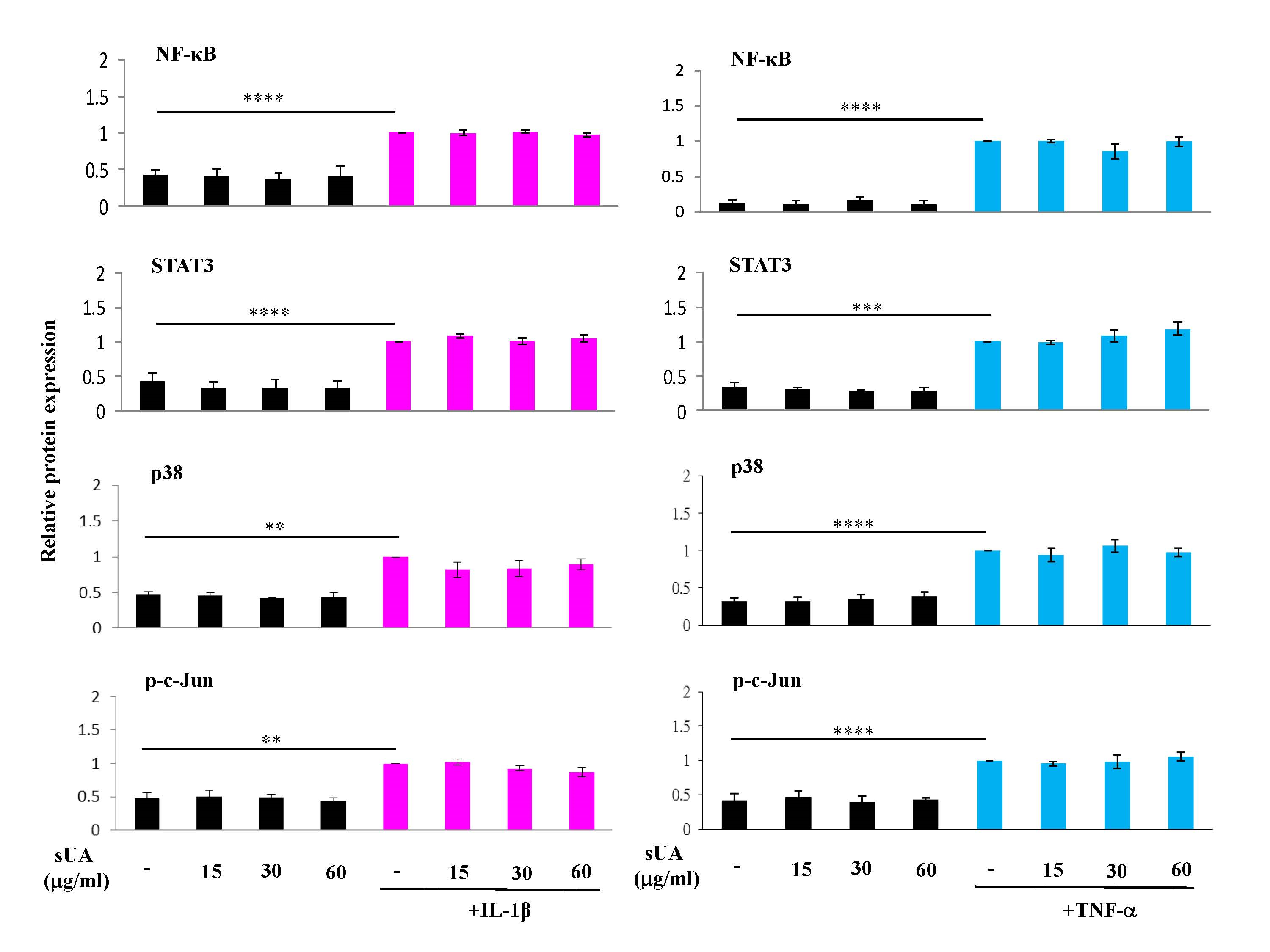


**Supplementary figure 3** Statistical analysis of sUA effects on TNF-α– and IL-1–induced NF-κB, STAT3, p38 and p-c-Jun expression as shown in Fig. 3. The statistical results are from at least 3 independent experiments. *p < 0.05; **p < 0.01; ***p < 0.001; ****p < 0.0001.


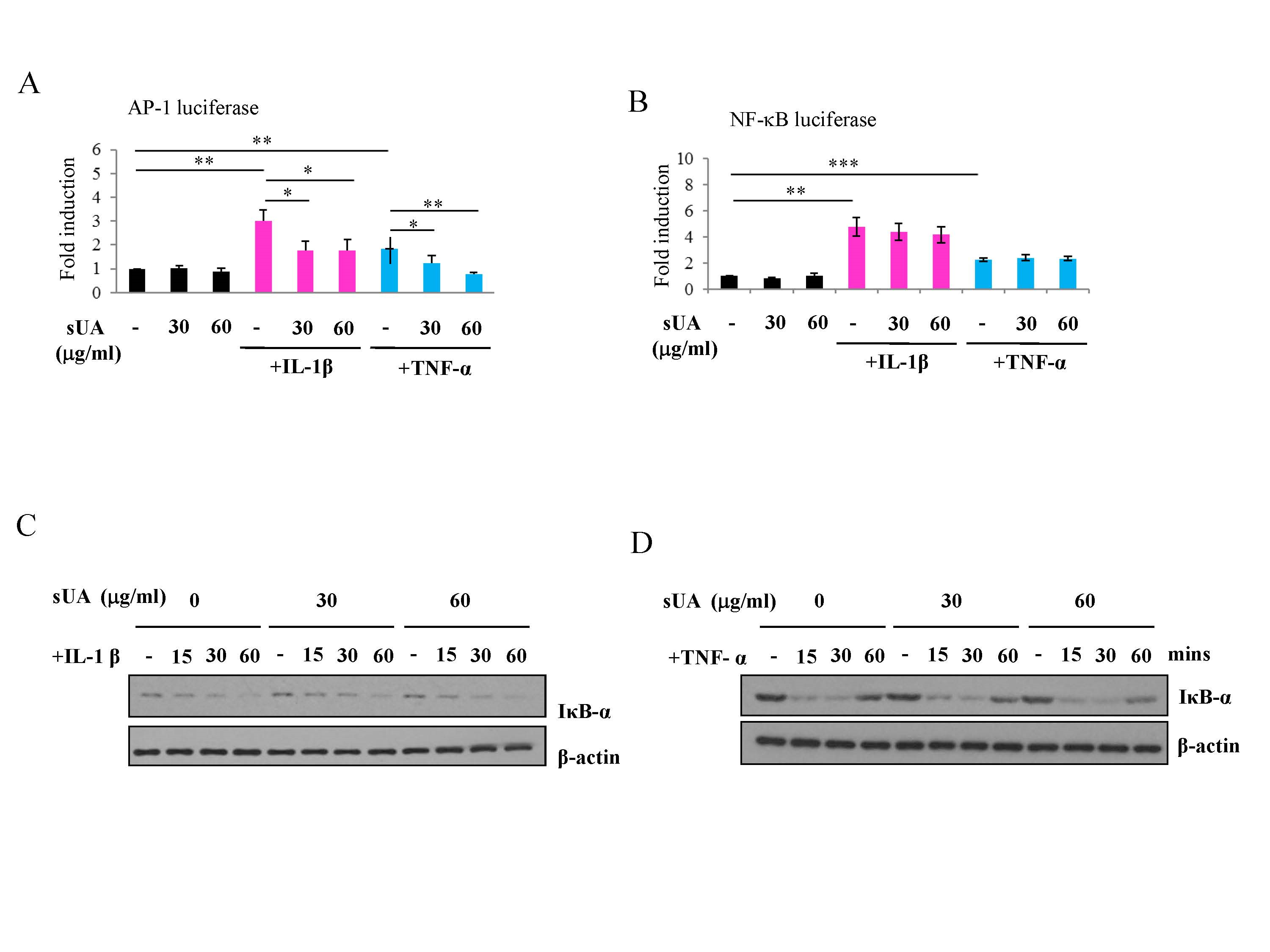


**Supplementary figure 4** Effects of sUA on activation of AP-1 and NF-κB stimulated with TNF-α– and IL-1 in porcine chondrocytes. Porcine chondrocytes (2 x 10^7^) were transfected with 15 μg reporter DNA (AP-1 or NF-κB) Trans IT transfection reagent mixture in Petri dish. Twenty-four h after transfection, the cells were washed, passed to 24-well plates and treated with different concentrations of sUA for 24 h, followed by stimulation with IL-1 or TNF-α for another 24 h. The total cell lysates were prepared and luciferase activity was determined (A and B, respectively). The effects of sUA in IL-1– and TNF-α–stimulated IκBα degradation were examined with Western blot (C and D, respectively). The statistical results are from at least 3 independent experiments. *p < 0.05; **p < 0.01; ***p < 0.001.


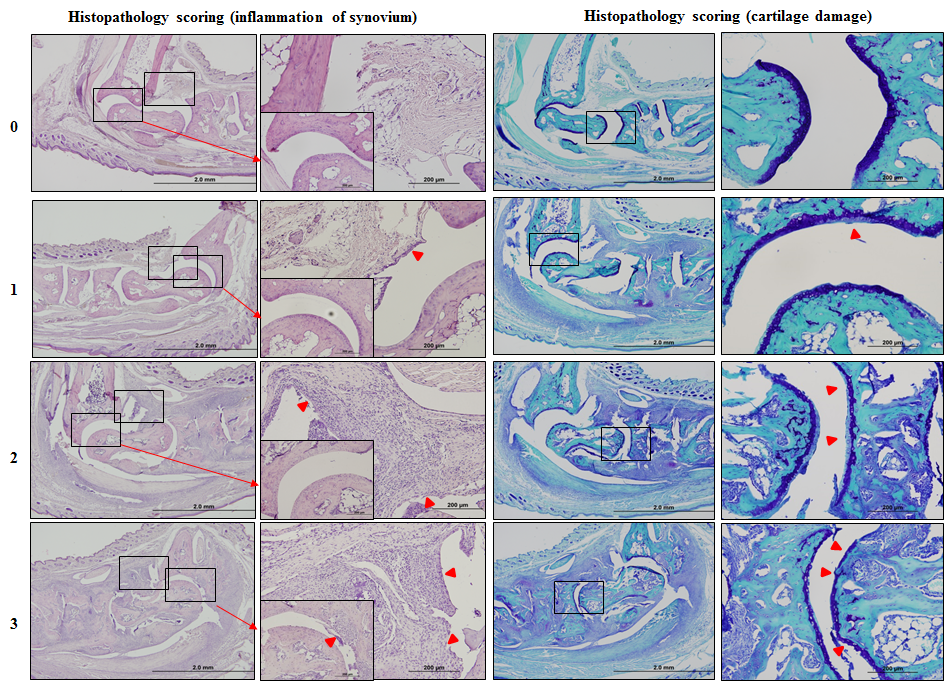


**Supplementary figure 5** Representative images showing the histopathology scoring for synovium inflammation and cartilage damage (red triangles). Scoring was performed according to the description by other researchers.


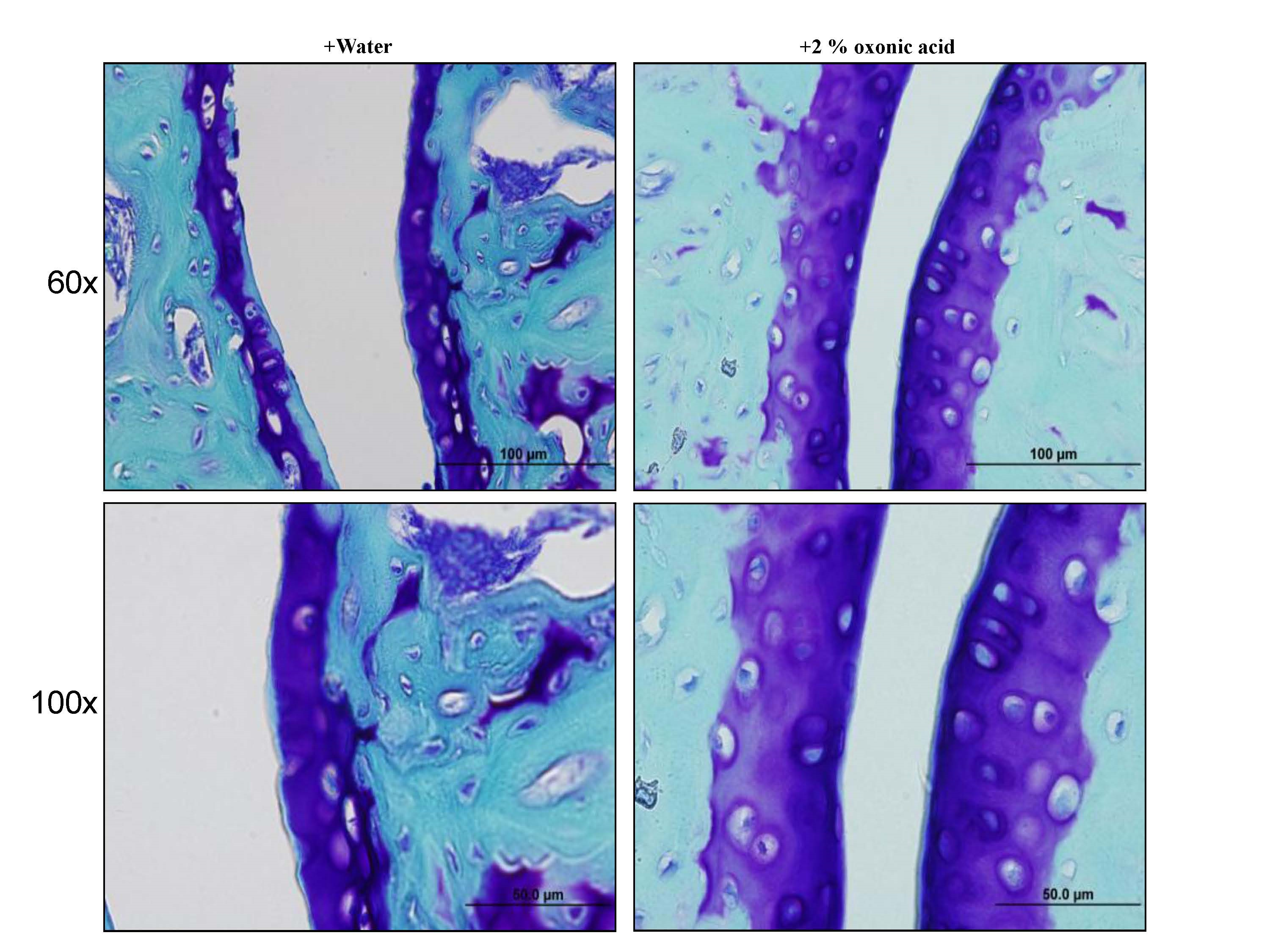


**Supplementary figure 6** The 60x and 100x magnification images for Fig. 6E.


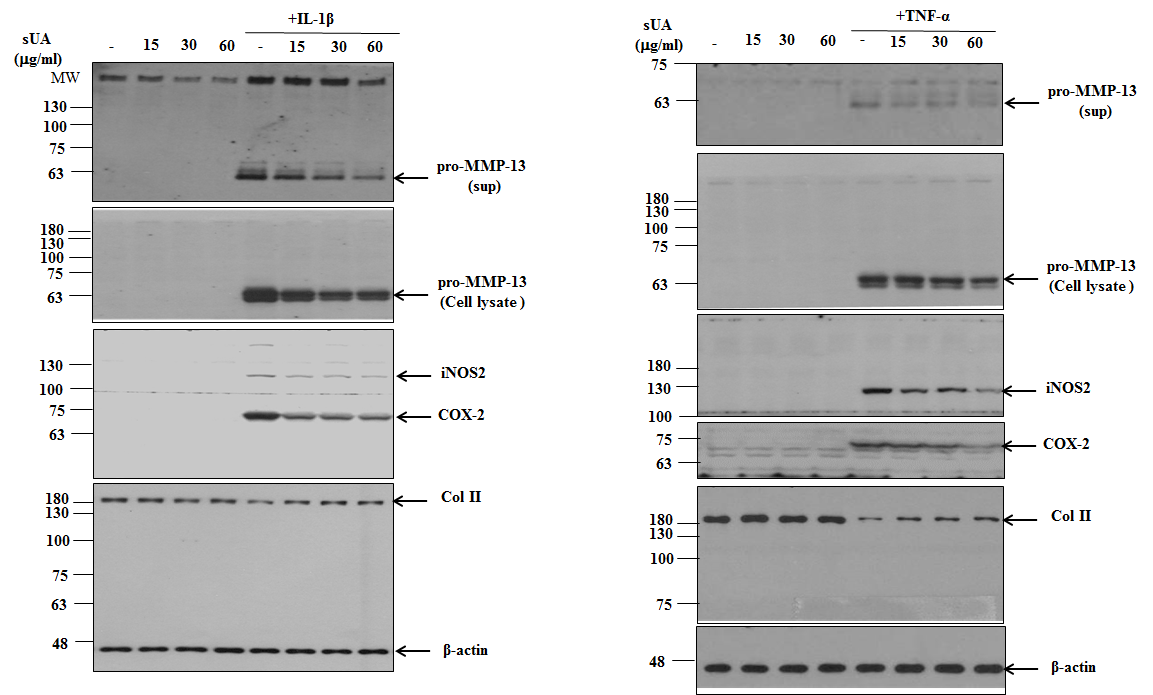


**Supplementary figure 7** The full-length blots of the cropped pictures shown in Fig. 1.

A


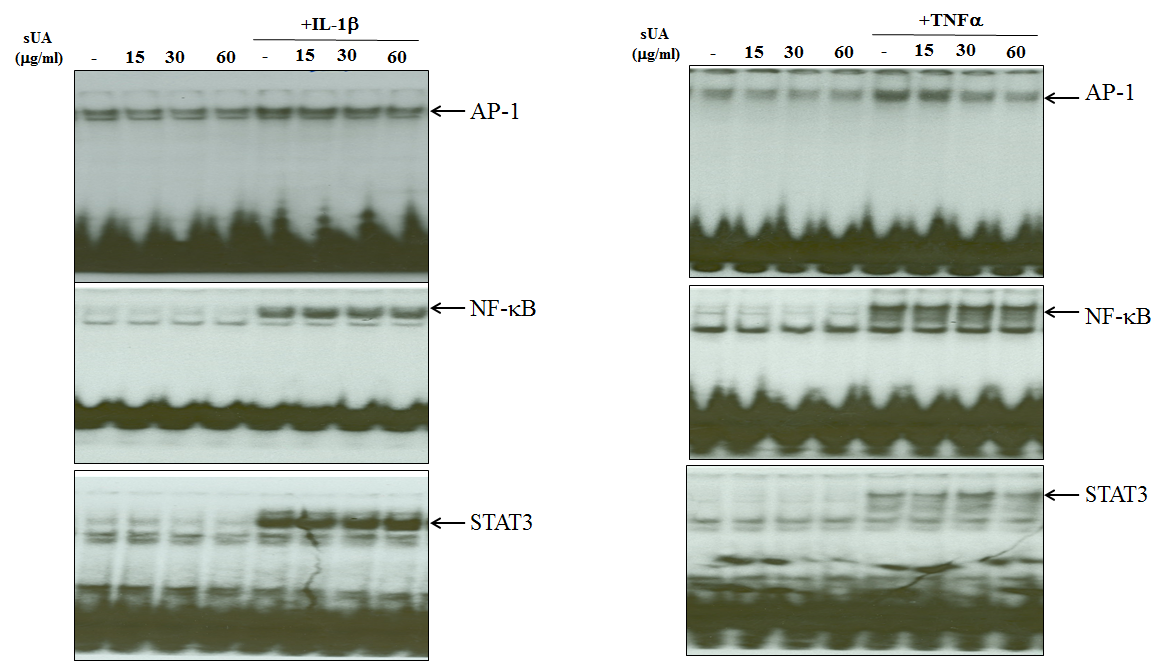


B


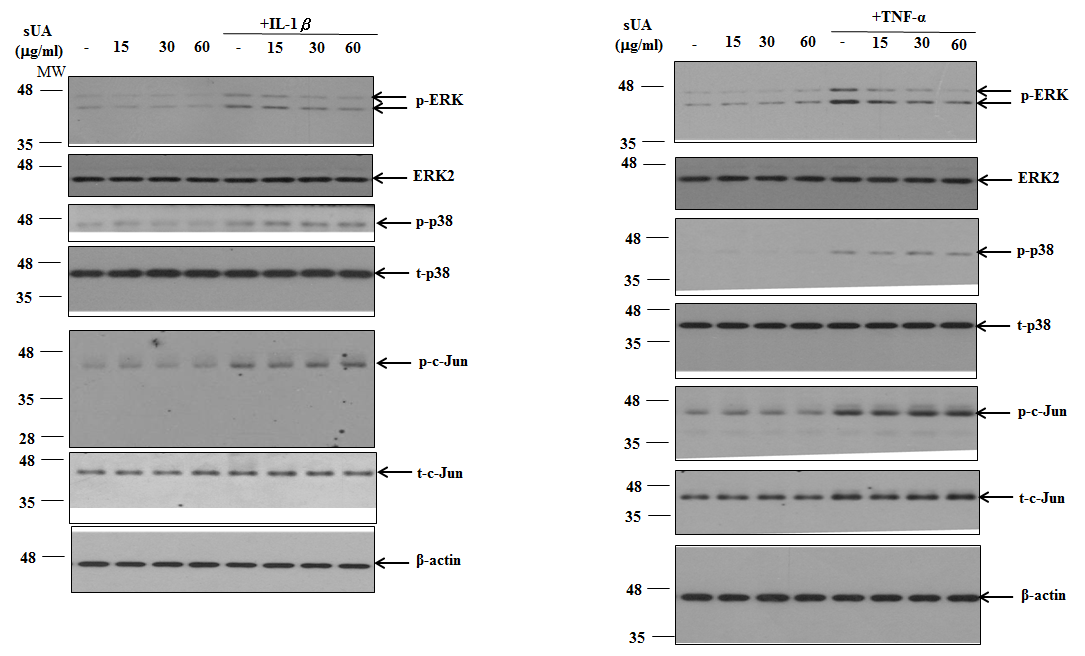


**Supplementary figure 8** The full-length blots of the cropped pictures shown in Fig. 3A and B.


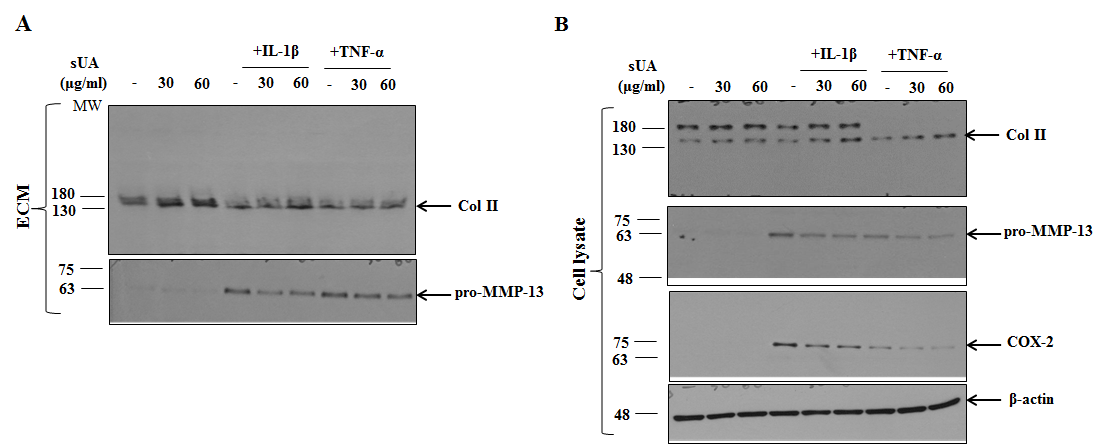


**Supplementary figure 9** The full-length blots of the cropped pictures shown in Fig. 4A and B.
